# Modulation of Phytoalexin Biosynthesis in Engineered Plants for Disease Resistance

**DOI:** 10.3390/ijms140714136

**Published:** 2013-07-08

**Authors:** Philippe Jeandet, Christophe Clément, Eric Courot, Sylvain Cordelier

**Affiliations:** Laboratory of Stress, Defenses and Plant Reproduction, Research Unit “Vines and Wines of Champagne”, UPRES EA 4707, Faculty of Sciences, University of Reims, P.O. Box 1039, Reims 51687, France; E-Mails: christophe.clement@univ-reims.fr (C.C.); eric.courot@univ-reims.fr (E.C.); sylvain.cordelier@univ-reims.fr (S.C.)

**Keywords:** plant engineering, phytoalexins, transcriptional factors, hormones, elicitors, regulation

## Abstract

Phytoalexins are antimicrobial substances of low molecular weight produced by plants in response to infection or stress, which form part of their active defense mechanisms. Starting in the 1950’s, research on phytoalexins has begun with biochemistry and bio-organic chemistry, resulting in the determination of their structure, their biological activity as well as mechanisms of their synthesis and their catabolism by microorganisms. Elucidation of the biosynthesis of numerous phytoalexins has permitted the use of molecular biology tools for the exploration of the genes encoding enzymes of their synthesis pathways and their regulators. Genetic manipulation of phytoalexins has been investigated to increase the disease resistance of plants. The first example of a disease resistance resulting from foreign phytoalexin expression in a novel plant has concerned a phytoalexin from grapevine which was transferred to tobacco. Transformations were then operated to investigate the potential of other phytoalexin biosynthetic genes to confer resistance to pathogens. Unexpectedly, engineering phytoalexins for disease resistance in plants seem to have been limited to exploiting only a few phytoalexin biosynthetic genes, especially those encoding stilbenes and some isoflavonoids. Research has rather focused on indirect approaches which allow modulation of the accumulation of phytoalexin employing transcriptional regulators or components of upstream regulatory pathways. Genetic approaches using gain- or less-of functions in phytoalexin engineering together with modulation of phytoalexin accumulation through molecular engineering of plant hormones and defense-related marker and elicitor genes have been reviewed.

## 1. The Concept of Phytoalexins

The concept of phytoalexins, that is antimicrobial substances of low molecular weight produced by plants in response to infection or stress, was proposed in 1940 by Müller and Börger [1] in the study of the interaction between potato and *Phytophthora infestans*. Briefly, the inhibition of the development of a race of *P. infestans* which normally develops profusely on potato tubers following infection of the potato tubers with another race of *P. infestans* able to initiate hypersensitive reaction (HR), was caused by a “principle” produced by the hypersensitively-reacting plant cells. The principle was named a phytoalexin. The term phytoalexin was derived from Greek to mean “warding-off agents in plants” and the proposal of this elegant concept was made after deliberating two important phenomena in plant pathology. The first one is the active response of the plant cell to attempted infection; the second one is the acquisition of resistance by plants after exposure to microorganisms [2].

A fundamental feature of this definition is that it restricts phytoalexins to compounds which are produced from remote precursors, through *de novo* synthesis of enzymes. At the time they have been discovered, phytoalexins were, therefore, clearly distinguished from other antimicrobial substances which are pre-formed or arise directly from inactive precursors [3]. As a corollary, the fact that these compounds constitute a defense response of plants to biotic and abiotic stresses, makes the regulatory networks involved in their induction extremely complex. For instance, studies on the signaling pathways controlling the induction of camalexin, the major phytoalexin from *Arabidopsis*, particularly underline the complexity of these regulatory mechanisms, which in turn depend on the infecting pathogen [4]. From reports evaluating the response of *Arabidopsis*-jasmonic acid (JA) mutants to *Alternaria brassicicola* it ensued that synthesis of this phytoalexin was not JA-dependent [5,6]. Conversely, JA has been found to form part of the regulatory pathways for camalexin biosynthesis in the response of that plant to *Botrytis cinerea* [7]. In fact, absence of functional jasmonate signaling in *Arabidopsis* mutants resulted in dramatic differences in phytoalexin accumulation upon infection by *B. cinerea*. Other results showed that the regulation of camalexin production can be controlled by salicylic acid (SA)-independent [8,9] or SA-dependent [10] signaling pathways. Camalexin production was also found to be decreased in response to bacterial or fungal infection in *Arabidopsis* mutants impaired in ethylene signaling [5,11]. Suppression of auxin signaling in *Arabidopsis* re-directed secondary metabolism towards glucosinolates and away from camalexin [12] (see section 4). Recent works have reported a regulation of camalexin biosynthesis involving two Mitogen-Activated Protein Kinases (MAPKs), inducing in turn the responses further downstream, especally camalexin accumulation [13] (see section 5). The two transcriptions factors WRKY40 and WRKY18 were also found to be involved in the regulation of some enzymes of the camalexin pathway, namely regulation of the cytochrome P450 monoxoygenase 71A13 catalyzing transformation of indole acetaldoxime to indole-3-acetonitrile on the route to camalexin [14]. Based on this example, it thus appears that regulatory pathways for phytoalexin induction are complex. Modulation of phytoalexin biosynthesis in engineered plants for disease resistance typically reflects this complexity.

Phytoalexins form part of the defense mechanisms of plants against phytopathogenic microorganisms. These are antibacterial, antifungal and antiviral compounds showing phytotoxic activities as well. It is now clear that phytoalexins exhibit toxicity across much of the biological spectrum, prokaryotic and eukaryotic, and that their activities are by no means confined to phytopathogenic fungi though considerable work has been done on plant/fungus interactions [15].

Are phytoalexins active? Relatively ancient studies reporting on the direct comparison of the protectant activity of isoflavonoid phytoalexins and the fungicides benomyl and mancozeb, have shown that phytoalexins are considerably less toxic than chemical fungicides [16]. Although differences in activity do occur between and within phytoalexin families, effective doses of phytoalexins generally fall within order of magnitude 10^−5^ to 10^−4^ M [17–19]. Beside their fungistatic action, phytoalexins can exert some fungitoxic activities as their action results in cytological abnormalities in fungal cells. Resveratrol and pterostilbene, for instance, two phytoalexins from grapevine, induce the formation of curved germ tubes, cessation of growth of some germ tubes with protoplasmic retraction in the dead hyphal tip cell, cytoplasmic granulation of the cellular content, disorganization of mitochondria and disruption of the plasma membrane in *B. cinerea* conidia [17–19]. Phytoalexins may also act as uncoupling agents of electron transport and photophosphorylation, leading to a rapid and complete cessation of respiration in *B. cinerea* conidia [17] and are able to induce fungal apoptotic-like programmed cell death as a result of the action of camalexin on *B. cinerea* [20].

The question as to whether phytoalexins are produced by all plants is remaining as there are few reports of thorough examinations concluding that these compounds are not synthesized by a particular plant species [3]. Even today too few families have been studied to permit the assertion that phytoalexin synthesis is a universal defense mechanism of plants [2]. Knowledge on phytoalexins from 1960 to 1980 was based essentially on extensive works in two plant families, Leguminosae or Fabaceae [21] and Solanaceae [22], and investigations of one or of a few species in each of a number of other plant families (Vitaceae, Poaceae, Malvaceae, Euphorbiaceae, Moraceae, Orchidaceae, Ginkgoaceae…) [4,23].

**Figure 1:**
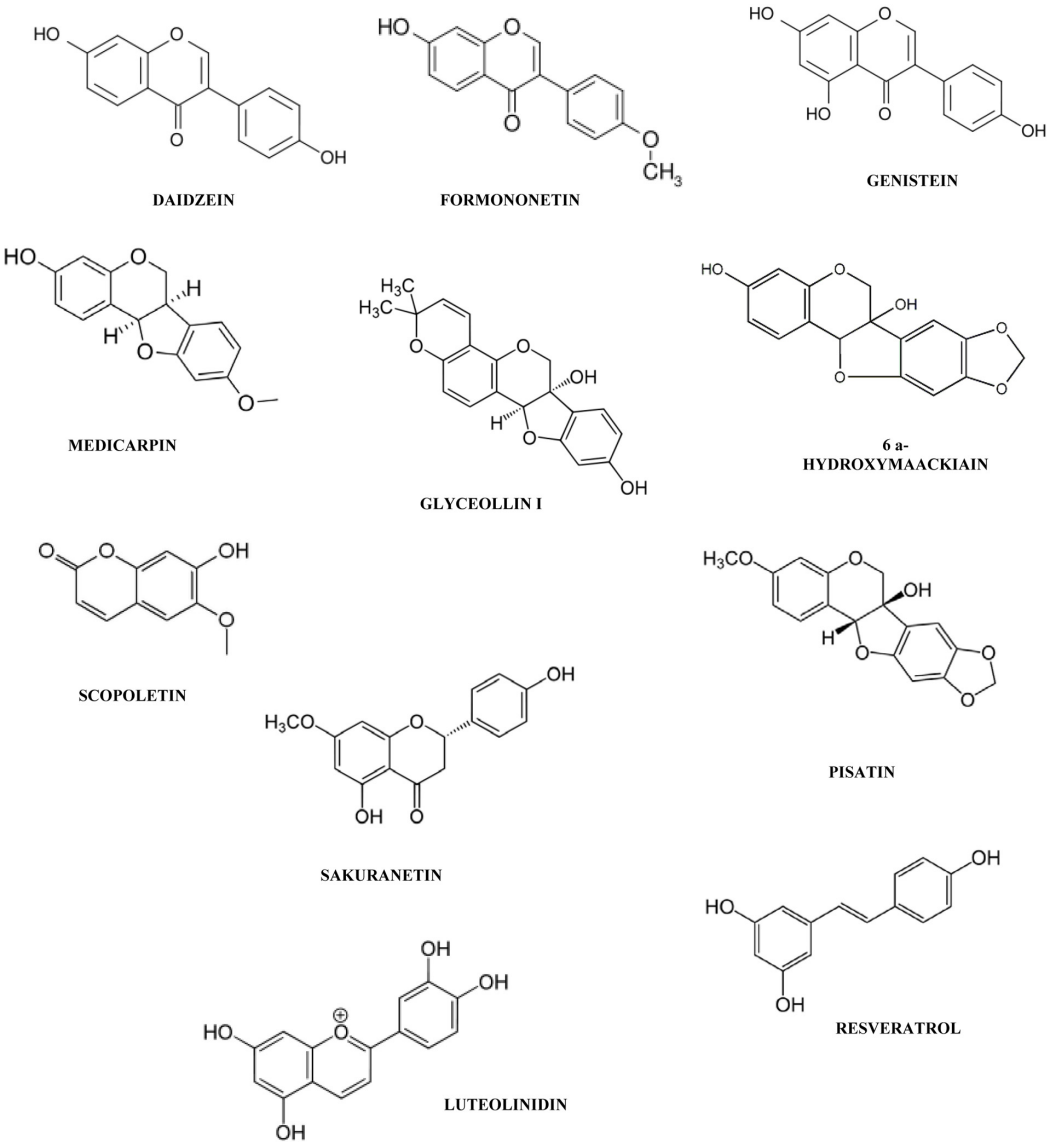
Chemical structures of some phytoalexins cited in this work: daidzein, formononetin, sakuranetin and genistein (simple isoflavones); medicarpin, maackiain, pisatin and glyceollin I (pterocarpans); resveratrol (stilbenes); scopoletin (coumarins); luteolinidin (3-desoxyanthocyanidins).

**Figure 2:**
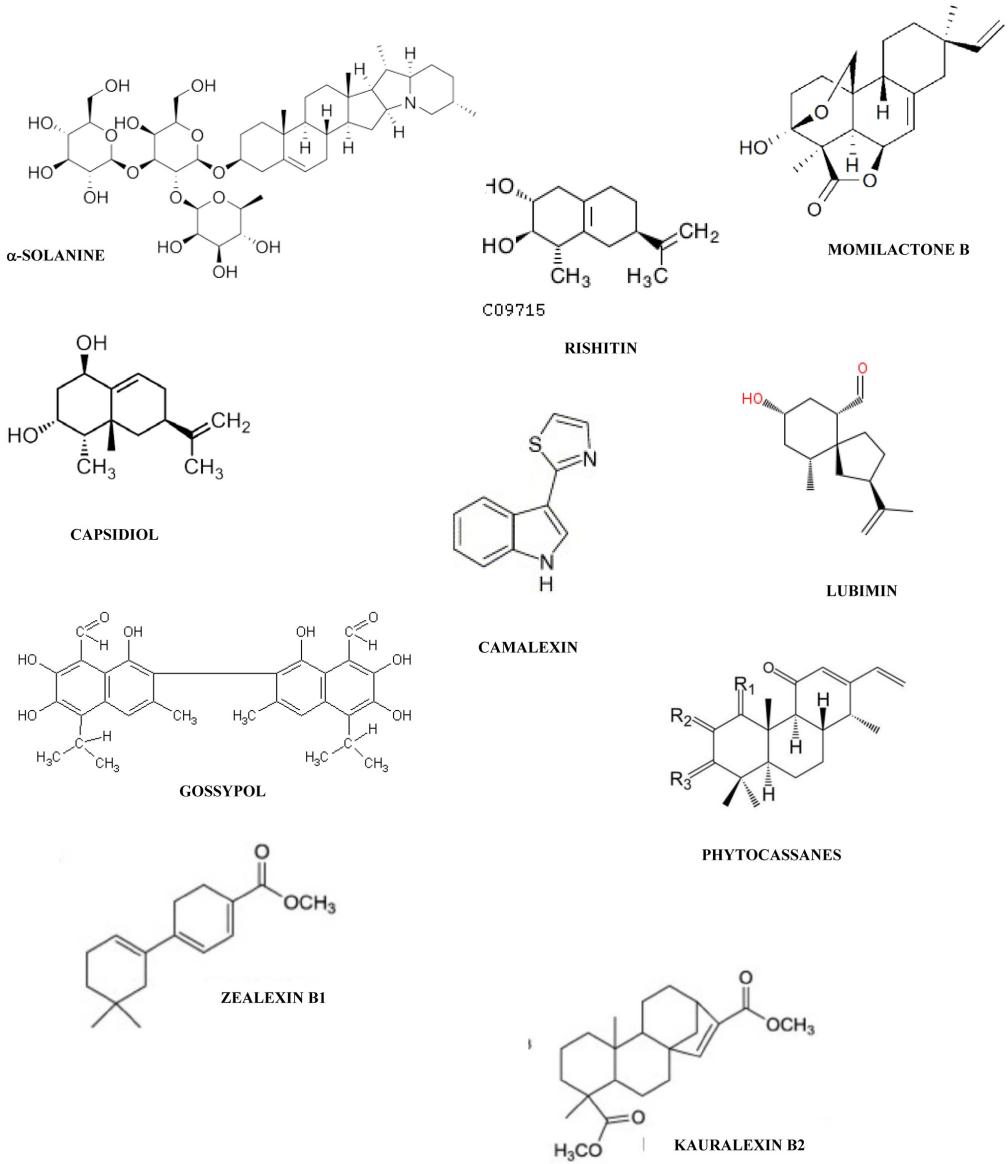
Chemical structures of some phytoalexins cited in this work: α-solanine (steroid glycoalkaloids); rishitin, lubimin, capsidiol, gossypol, kauralexin B2; zealexin B1 and momilactone B (norsesquiterpenoids/sesquiterpenoids); and phytocassanes.

Phytoalexins display an enormous chemical diversity. The most studied members of the phytoalexins from Leguminosae (Fabaceae) are the simple isoflavones, daidzein [24], formononetin [25], and genistein [26]; the complex isoflavone, wighteone [27]; the simple isoflavanones, vestitone [28] and kievitone [29]; the pterocarpans, medicarpin [30], maackiain [31], pisatin [31], phaseolin [32] and glyceollin [33]; the simple isoflavan, vestitol [34]; the simple coumestan, coumestrol [35]; the furanoacetylenes, wyerone acid [36] and wyerone [37] and stilbenes [38] (Figure 1). Four main classes of phytolexins have been reported in Solanaceae: the phenylpropanoid-related phytoalexins caffeic acid and chlorogenic acid [39]; the steroid glycoalkaloids, α-solanine and α-chaconine [40]; the norsesquiterpenoids/sesquiterpenoids, rishitin [41,42] lubimin [43], capsidiol [44] and the coumarin, scopoletin [45] (Figures 1 and 2). Maize and rice are Poaceae crop plants. The main phytoalexins in rice are the two 9-β-pimaradiene diterpenes, momilactones A and B [46], the flavanone, sakuranetin [47] and the diterpenes, phytocassanes A–E [48]. Two new phytoalexin families have recently been discovered in maize: the *ent*-kaurane-related diterpenoids, kauralexins [49] and the acidic sesquiterpenoids, zealexins [50] (Figures 1 and 2). The 3-deoxyanthocyanidins including luteolinidin (Figure 1) and apigeninidin, form an unusual group of flavonoid phytoalexins produced by members of the Poaceae, for instance, as a response of sorghum mesocotyls to infection by pathogenic and nonpathogenic fungi [51–53]. Gossypol and its congeners, that is, naphthaldehyde compounds, constitute the main phytoalexins from cotton plants (*Gossypium* spp.) of the Malvaceae family [54]. We will end this list, which is far from being exhaustive, with camalexin, the major phytolexin from the Brassicaceae (also known as Cruciferae) [55] and resveratrol, the major phytoalexin from the Vitaceae [56] (Figures 1 and 2).

How can our knowledge of phytoalexins be used to develop new approaches to plant disease control? Phytoalexins have long been recognized to as being important in the defense mechanisms of plants against phytopathogenic microorganisms. Their potential biological properties have stimulated a ferment of activity concerning the biosynthesis and the metabolism of these compounds in the plant. Research has gone forward because these compounds are thought to help agriculturally and economically important crop plants withstand colonization by pathogens. In many instances, a close correlation has been found between phytoalexin production and resistance to diseases.

Use of modern molecular biology tools for elucidating the control mechanisms of phytoalexin synthesis and for engineering disease resistant plants is based on the expression of stress- or disease-related genes [57]. Genetic engineering of phytoalexins for disease resistance requires manipulation of a single or a few genes directly involved in their biosynthetic pathways or involved in their signaling/regulatory pathways [58]. We will describe in this review, efforts that have been done for engineering phytoalexin production in plants as well as provide information on how phytoalexin accumulation can be modulated through engineering of plant hormones, defense-related markers or elicitors.

## 2. Genetic Manipulation of Phytoalexin Production and Disease Resistance Addressing Gain- or Loss-of-Function Genetic Approaches

As surprising as it may seem, there have been not so much reports of attempts to manipulate phytoalexins by genetically modifying expression of biosynthetic pathway genes for disease resistance in plants. Works in this area address both gain- or loss-of-functions genetic approaches, with the most abundant literature concerning resveratrol, the major phytoalexin from Vitaceae (for reviews see [59–63]).

**Figure 3:**
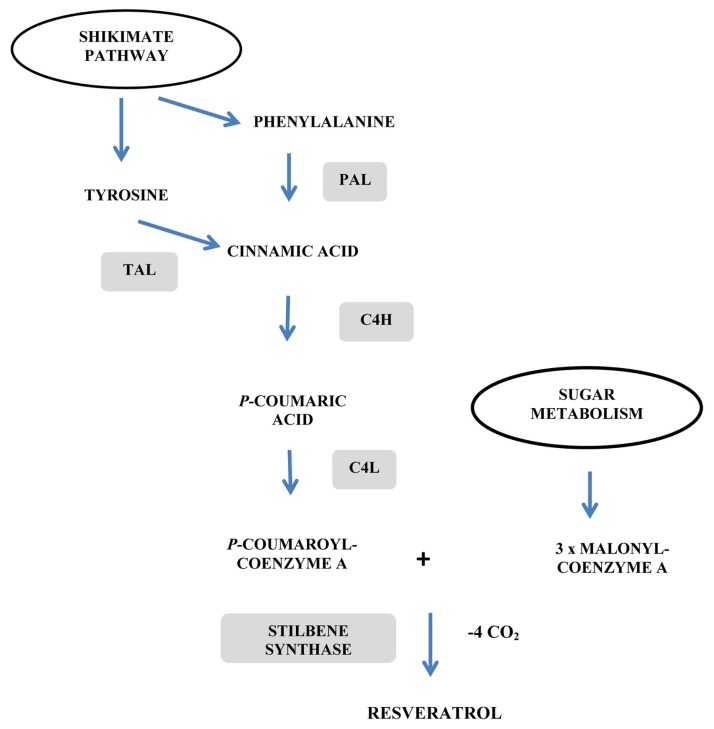
*In vivo* biosynthesis of resveratrol from phenylalanine or tyrosine via the phenylpropanoid/polymalonate pathway. PAL/TAL: phenylalanine/tyrosine ammonia lyase; C4H: cinnamate-4-hydroxylase; C4L: coumarate:coenzyme A ligase; STS: stilbene (resveratrol) synthase.

Resveratrol is obtained through the universal phenylpropanoic acid pathway beginning with phenylalanine (via the phenylalanine ammonia lyase, PAL) or with tyrosine (via the tyrosine ammonia lyase, TAL) (Figure 3). The obtained cinnamic acid is activated by ligation to coenzyme A by 4-coumaroyl:CoA ligase (C4L). Resveratrol is then synthesized by the stilbene synthase (STS) which acts to condense three successive units of malonyl-CoA with *p*-coumaroyl-CoA. STS, the key enzyme of resveratrol synthesis, thus uses as substrates precursor molecules that are present throughout the plant kingdom. Therefore, the introduction of a single gene is sufficient to synthesize resveratrol in heterologous plant species. Transformations have been then operated to investigate the potential of stilbene biosynthetic genes to confer resistance to pathogens. Interest in resveratrol engineering has increased due to its antimicrobial properties acting as an allelochemical or a phytoalexin, but also due to its antioxidant and antitumor capabilities (for reviews see [59–64]). Transfer experiments began with two grapevine STS genes (*Vst1* and *Vst2*) introduced into tobacco under the control of the stress responsive promoter pVst1, conferring that plant a higher resistance to *B. cinerea* infection [65]. This study constituted the first report of a disease resistance resulting from foreign phytoalexin expression in a novel plant. In the same way, Hipskind and Paiva [66] have transformed alfalfa (*Medicago sativa*) with the *Arachis hypogea* STS *AhRS* gene transcriptionally regulated by an enhanced cauliflower mosaic virus (CaMV) 35S promoter, conferring increased resistance to *Phoma medicaginis*. The grapevine rootstock 41-B overexpressing the grapevine *Vst1* stilbene synthase gene under the control of the fungus inducible promoter *ms* PR 10.1 produced high stilbene levels and exhibited *in vitro* resistance to *B. cinerea* [67]. Similar transformations have improved the resistance of rice to *Piricularia orizae* (grapevine STS gene *Vst1* with the stress responsive promoter pVst1) [68], of barley and wheat to *B. cinerea* and to powdery mildew (grapevine STS gene *Vst1* with the stress responsive promoter pVst1 + CaMV 35S) [69,70], of tomato to *P. infestans* (two grapevine STS genes *Vst1* and *Vst2* under the control of the stress responsive promoter pVst1) [71], of wheat to *Puccinia recondita* and *Septoria nodorum* (two grapevine STS genes *Vst1* and *Vst2* with the stress responsive promoter pVst1 + CaMV 35S) [72], of papaya to *Phytphthora palmivora* (grapevine STS gene *Vst1* with the stress responsive promoter pVst1) [73] and of *Rehmannia glutinosa* to *Fusarium oxysporum* with the *Arachis hypogea* STS *AhRS3* gene transcriptionally regulated by the constitutive CaMV 35S promoter [74]. More recently, overexpression of a resveratrol gene *PcRS* from *Polygonum cuspidatum* under the control of the constitutive CaMV 35S promoter in transgenic *Arabidopsis* was shown to result in the accumulation of *trans*-piceid (the 3-*O*-β-d-resveratrol glucoside) and an increased resistance to the fungal pathogen, *Colletotrichm higginsianum* [75].

All these results show that resveratrol is a determinant factor in the expression of resistance of plants to phytopathogens and underline the above-mentioned interest for engineering this phytoalexin in plants. However, there were some cases for which no resistance was observed after transforming the plants with *STS* genes. For example, transformation of white poplar (*Populus alba*) with the grapevine *StSy* or with a pinosylvin-synthase-encoding gene from *Pinus sylvestris* did not confer any increased resistance to *Melaspora pulcherrina* (rust disease) [78,79]. Similarly, no increased resistance against *B. cinerea* has been observed in STS transgenic kiwi plants expressing the grapevine STS *pSV25* under the control of pCAMV 35S [80]. These contradictory results suggest that pathogen control by transgenic STS plants can be significant but may be considered as empiric and not predictable. Generally, STS expression does not trigger any detrimental effect on plant development, growth, morphology or fertility. However, in few cases, STS overexpression can lead to morphological alterations in transgenic plants such as color modification and male sterility as observed in tobacco and petunia [81] or in conifers [82]. Such events may be explained by the possible transgene insertion site, such as, for instance, insertions within a gene involved in male fertility for sterile lines.

**Figure 4:**
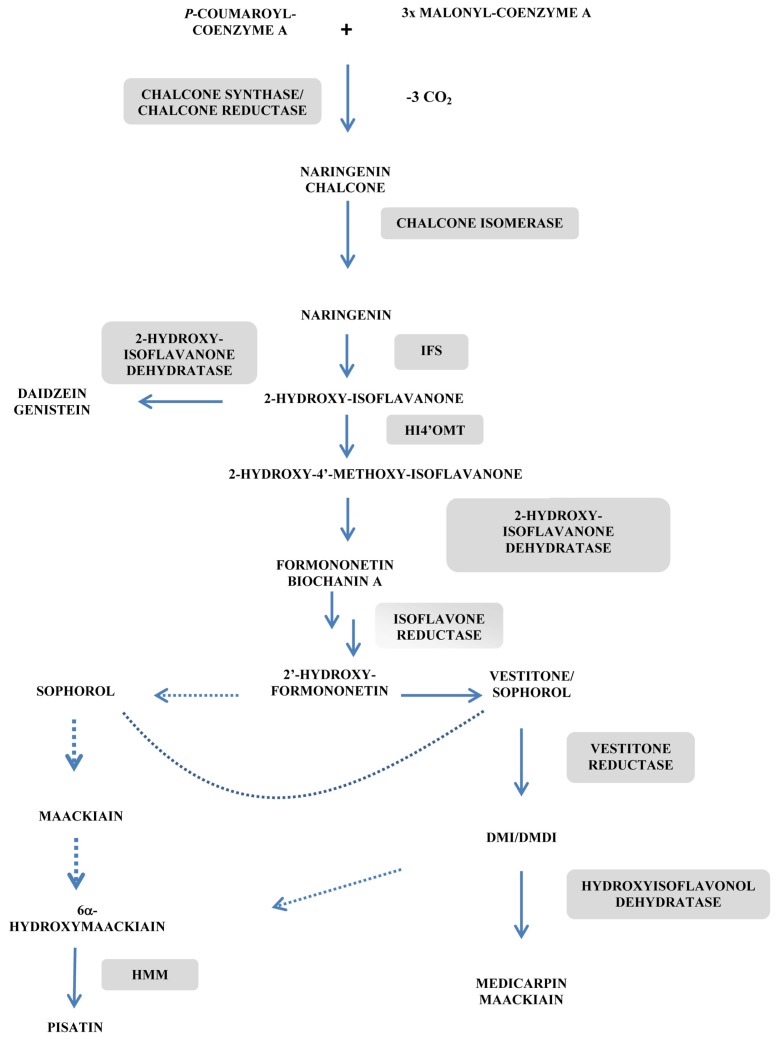
Scheme of biosynthetic pathways to the main phytoalexins from Leguminosae. IFS: 2-hydroxy isoflavanone synthase; DMI: 7,2′-dihydroxy-4′-methoxy-isoflavanol; DMDI: 7,2′-dihydroxy-4′,5′-methylenedioxy-isoflavanol; HMM: 6α-hydroxymaackiain 3-*O*-methyltransferase; HI4′OMT: SAM: 2,7,4′-trihydroxy-isoflavanone 4′-*O*-methyltransferase (adapted from [76,77]). The dashed arrows represent hypothetic steps and the solid arrows denote reactions for which the catalyzing enzymes have been cloned.

Isoflavonoids constitute the second major phytoalexin family that has attracted considerable attention for plant engineering though this gain of interest was due mainly to the antioxidant and anticancer activities of these compounds. 4′-*O*-methylation of isoflavonoid-type compounds is a prerequisite for further substitutions of the isoflavonoid core, leading to the pterocarpan phytoalexin medicarpin. Genetic manipulation of the isoflavone 7-*O*-methyltransferase has been reported in alfalfa (*Medicago sativa*) in relation to increased resistance of that plant to *Phoma medicaginis*, the causal agent for spring black stem and leaf spot disease [83]. Gene constructs harboring the full-length isoflavone-*O*-methyltransferase from alfalfa (IOMT8) cDNA in the sense and antisense orientations under control of the constitutive promoter 35S were obtained. Unexpectedly, overexpression of IOMT led to 7-*O*-methylisoflavonoid derivatives *in vitro* as well as *in vivo* unchallenged leaves of transgenic alfalfa when ectopically expressed but mainly produced 4′-*O*-methylderivatives, such as formononetin (4′-*O*-methyldaidzein) and medicarpin in plants elicited with metallic cations or infected with *P. medicaginis*. Such apparently different regiospecificities of IOMT expressed *in vivo* or *in vitro* will not be discussed further but underlined potential metabolic channeling at the entry point into the isoflavonoid-type phytoalexin pathway (Figure 4).

In antisense IOMT transgenic lines, root tissues from those plants exhibited markedly reduced phytoalexin concentrations as a consequence of a potential downregulation of the isoflavonoid content. Ectopically overexpression of IOMT in unchallenged transgenic leaves produced 7-*O*-methylisoflavonoids but no 4′-*O*-methylisoflavonoids (see above). Conversely, abiotic elicitation using spores of *P. medicaginis* led to the accumulation of the phytoalexins formononetin glucoside and medicarpin both in empty-vector control plants and IOMT over-expressing plants 2 days post infection (dpi), being the content of medicarpin 2.5 fold higher in IOMT overexpressing lines (67 μg/g fresh WT *vs.* 30 μg/g fresh WT). Interestingly, high medicarpin accumulation rates in IOMT-overexpressing plants were associated with a marked reduction in the size of the brown *P. medicaginis* lesions on leaves measured 5 dpi (around 60% decrease as compared to empty-vector control plants), being this reduction of the disease symptoms well correlated with postinoculation increases in medicarpin concentrations, confirming the role of this compound in disease resistance in alfalfa. In contrast, authors failed to demonstrate any increased susceptibility in antisense IOMT lines with decreased amounts of medicarpin, as this may be the result of an incomplete inhibition of accumulation of the phytoalexin in those lines. Such a situation was not observed in *Arabidopsis* mutants deficient in camalexin where susceptibility to the necrotrophic fungus *Alternaria brassicicola* is highly correlated with the absence of this phytoalexin [5]. These different behaviors underline limitations that can be encountered by using loss-of-function genetic approaches for the understanding of plant/pathogen interactions.

In a similar approach, biosynthesis of the simple isoflavone genistein glucoside was engineered in leaves of alfalfa by constitutive expression of isoflavone synthase from *Medicago truncatula* [76] (Figure 4). The Mt*IFS1* cDNA from *M. truncatula* was subcloned into the pRTL2 vector and the expression cassette containing Mt*IFS1* flanked by CaMV 35S promoter and terminator sequences was cloned into the *Hin*dIII site of the binary vector pCAMBIA 2300. Mt*IFS1* was highly expressed in all organs, while genistein accumulation was limited to leaves. Other simple isoflavone-type phytoalexins were also found such as daidzein, biochanin A (4′-*O*-methylgenistein) and 3′-hydroxybiochanin A (pratensein) in transgenic lines with constitutive expression of Mt*IFS1*. Additionally, together with daidzein, other 5-deoxyflavone phytoalexins accumulated, for instance formonotenin, in transgenic lines irradiated with UV-B for 6h or as a result of plant infection with *P. medicaginis*. The pterocarpan phytoalexin, medicarpin, was also found to be accumulated, likely in relation to its antifungal activity [84]. MtIFS1-expressing plants contained much higher concentrations of formononetin and medicarpin than the vector control lines, being the levels of formononetin between 46 and 77 μg/g fresh WT in leaves 24 h to 72 h after infection, that is, concentrations able to inhibit the pathogen development.

Loss-of-function approaches were applied to study the role of the phytoalexin pisatin in the disease resistance of hairy roots of pea (*Pisum sativum* L.) to the fungal pathogen *Nectria haematococca* through the genetic manipulation of enzymes involved in both the biosynthesis and the catabolism of this compound [85] (Figure 4). Six genetic constructs were thus designed: sense and antisense-oriented cDNAs of isoflavone reductase (IFR), (+) 6α-hydroxymaackiain 3-*O*-methyltransferase (HMM) fused to the 35S CaMV promoter, being these two enzymes involved in pisatin biosynthesis, and a pisatin demethylating activity (PDA) from *N. haematococca*. Reduced amounts of pisatin were observed in hairy root cultures transformed with antisense-*Hmm* which led to fewer *Hmm* transcripts, resulting in less HMM protein and enzyme activity in response to elicitation with CuCl_2_ or infection with the pea-pathogenic fungus, *N. haematococca*. As a consequence of the transformation with antisense *Hmm*, roots became more susceptible to *N. haematococca*. As expected, cultures with the lowest levels of pisatin were those containing the pisatin demethylating activity. At the time this paper was published, it appeared to be the first case of producing transgenic plant tissues with reduced capability to accumulate a given phytoalexin and demonstrating that such a tissue was less resistant to fungal infection [85]. Additionally, this loss-of-function genetic approach afforded direct evidence of the role of pterocarpan phytoalexins in plant defense mechanisms [77].

One of the most highly characterized systems for the delineation of the roles of secondary metabolites in plant disease resistance is the model pathosystem soybean (*Glycine max*)-*Phytophthora sojae*. An RNAi silencing approach was used to illustrate the role played by 5-deoxyisoflavonoid phytoalexins in the resistance and hypersensitive cell death of soybean to *P. sojae* [86]. Silencing either isoflavone synthase (IFS) or chalcone reductase (CHR) led to suppression (90%) of 5-deoxyisoflavones (daidzein and its derivatives) and glyceollin as well as disease resistance to *P. sojae* upon treatment with a cell wall glucan elicitor (WGE) from this pathogen. Loss of resistance was accompanied by suppression of HR cell death. Thus, 5-deoxyisoflavonoid-type phytoalexins from soybean were shown to play a critical role in the establishment of HR and race-specific resistance. Interestingly a third gene silencing RNAi approach targeting the gene *PR-2* which encodes the elicitor-releasing endoglucanase, led to loss of HR, loss of disease resistance and most importantly to suppression of the phytoalexin response upon treatment with WGE. This underlined links existing between a general resistance elicitor (pathogen-associated molecular pattern), HR cell death, race-specific resistance and accumulation of phytoalexin compounds in a given plant—pathogen system.

Loss-of-functions approaches were also used for the study of the role of 3-deoxyanthocyanidin phytoalexins in the resistance of sorghum (*Sorghum bicolor*) to anthracnose leaf blight caused by the phytopathogenic fungus *Colletotrichum sublineolum* as well as for the delineation of the biosynthesis pathway of this particular group of phytoalexins [87]. In the sorghum, it was shown that the corresponding *yellow seed1* gene (*y1*) encoded a R2R3 type of MYB domain protein which regulates the coordinate expression of *chalcone synthase (chs1)*, *chalcone isomerase*, *dihydroflavonol reductase* and *flavonoid 3*′*-hydroxylase* [88,89] genes required for the biosynthesis of 3-deoxyflavonoids including 3-deoxyanthocyanidin phytoalexins [87,90,91]. Interestingly, loss-of-function alleles of *y1* were found to be deficient in the synthesis of 3-deoxyanthocyanidins, these plants exhibiting severe symptoms characteristic of the anthracnose disease [87]. This directly supported the involvement of a functional *y1* gene in the increased resistance of sorghum to *C. sublineolum* [87]. The upper stream of the biosynthesis pathway leading to 3-deoxyflavonoids, especially formation of luteoferol from apiforol, that is, the precursor of the phytoalexin luteoninidin, was also shown to be under the control of the *red aleurone 1* (*pr1*) gene which encodes a CYP450-dependent flavonoid 3′-hydroxylase in maize, by using a loss-of-function approach [89].

Finally, loss-of-function mutants have been extensively used for the understanding of the role of the phytoalexin camalexin in the model plant *Arabidopsis* upon challenge with various bacterial or fungal pathogens. Five non-allelic phytoalexin-deficient mutants of *Arabidopsis* producing reduced amounts of camalexin during inoculation with bacteria or with biotrophic fungi (*pad1-1*, *pad2-1*, *pad3-1*, *pad4-1* and *pad5-1*) have been identified [92–94]. Some studies focused on the phytoalexin *Arabidopsis* mutant pad3-1, showing that the effect of the *PAD* mutation on the level of camalexin production was dependent on the pathogen [5]. This mutant was indeed found not to display altered susceptibility with *Pseudomonas syringae* nor with the biotrophic fungi *Perenospora parasitica* and *Erysiphe orontii* [92–94]. In another work, the same mutant was reported to be markedly more susceptible than its wild-type parental line upon infection with *Alternaria brassicicola* but this was not the case in transgenic lines upon inoculation with *B. cinerea*. In the latter, camalexin was undetectable whereas a strong camalexin response was reported in wild-type plants [5]. In the non phytoalexin producer-mutants *pad3-1* which exhibited resistance to *B. cinerea*, it was suggested that effectors other than camalexin which are not disabled in the transgenes, especially JA-or ethylene-dependent pathogenesis-related genes such as *PR3* and *PR4*, were good determinants for disease resistance [5]. Despite the observed discrepancies within these loss-of-function genetic approaches, the use of *PAD* mutants offers interesting models for studying the role of camalexin in the defense response of *Arabidopsis* against pathogens.

## 3. Genetic Manipulation of Phytoalexin Glucosylation and Disease Resistance

Glucosylation of scopoletin, a coumarin phytoalexin from tobacco (*Nicotiana tabacum* L.), is a particular example illustrating the crucial role of plant UDP-Glc:phenylpropanoid glucosyltransferases (UGTs) in plant defense responses and their relevance to the more general context of plant/pathogen interactions [95,96]. Glucosylation indeed intervenes in the accumulation, storage, transport and water solubility of a wide range of compounds [97].

Based on two different and consecutive studies including an antisense strategy, on one hand [98], and a gain-of-function approach, on the other hand [99], Saindrenan and colleagues have underlined the role of glucosyltransferases in scopoletin accumulation in tobacco plants following inoculation with Tobacco Mosaic Virus (TMV). In the first approach, downregulation of the expression of two tobacco salicylic acid- and pathogen-inducible UDP-glucosyltransferase genes *Togt 1* and *Togt 2* (acting on scopoletin glucosylation) was obtained by introducing the complete coding sequence of Togt 1 in the antisense orientation downstream from the constitutive CaMV 35S promoter in tobacco [98]. Expectedly, TOGT downregulation resulted in a decrease in scopoletin UGT activity together with a decline in the contents of both the aglycone and the glucoside of scopoletin named scopolin. Scopolin accumulation was reduced by 70% to 75% in tobacco reacting hypersensitively to TMV.

The TOGT inhibition-mediated decrease in both the scopolin and scopoletin contents could have been the result of a feedback inhibition of upstream enzymes of the scopoletin biosynthesis. Antisensing *Togt 1* was found to have no effects on other enzymes involved in plant defense mechanisms such as *O*-methyltransferases of class II (COMT II) or acidic β-1,3-glucanases [98]. Interestingly, transgenic lines downregulated for TOGT showed a 63% increase in TMV lesion surfaces *vs.* control plants relative to changes in scopolin and the TMV-replicate inhibiting-phytoalexin, scopoletin. As the antisense strategy used previously left open the possibility of a non-specific downregulation of a similar glucosyltransferase or of enzymes involved in the biosynthetic route to scopoletin, a gain-of-function approach was needed to further confirm the role of this pathogen-inducible glucosyltransferase during the hypersensitive response of *N. tabacum* to TMV.

Clonage of the *togt 1* full-length cDNA was carried out in the sense orientation downstream the 35S CaMV promoter into the pFB8 vector [99]. Basal scopoletin UGT activity in lines harboring the sense orientation construct was about 3-fold higher than in vector control plants and the resulting accumulation of scopoletin and scopolin was found to be 2-fold higher. Acceleration of the development of TMV lesions in transgenic TOGT overexpressing lines, despite increases in the phytoalexin content, was thought to result in a better restriction of TMV, thus preventing virus spread. These results thus clearly demonstrated the involvement of TOGT in scopoletin glucosylation *in planta*, controlling the accumulation of this antiviral compound during the hypersensitive reaction of tobacco to TMV; they also suggested the overall relevance of metabolic engineering of glucosyltransferases in the outcome of plant/pathogen interactions. Conversely, TOGT activity for scopoletin glucosylation was reported to be moderately expressed upon cytokinine-mediated resistance of tobacco to *Pseudomonas Syringae* pv *tabaci* [100].

## 4. Modulation of Phytoalexin Accumulation through Engineering of Plant Hormones

The essential role played by phytohormones in the induction of plant defense responses has been reviewed elsewhere [101–109]. Defense responses against biotrophs and necrotrophs are classically based on two antagonistic pathways, SA signaling controlling defense against biotrophs, JA/ethylene signaling controlling defense against necrotrophs. Some aspects of the involvement of SA, JA/ethylene in phytoalexin induction have been discussed in the particular case of camalexin (see section 1). There are also some examples of phytoalexin accumulation being up- or downregulated by overexpression of other signaling molecules such as auxins, abscisic acid, cytokinins and gibberellins.

Auxin homeostasis is considered as one of the components participating in the regulation of plant defense responses [110,111]. In many instances, auxins appear to negatively regulate plant defense mechanisms. In the case of plant/virus interactions, there are some examples relating auxin depletion and pathogen resistance [112–114]. Overexpression of *OsGH3.8*, a member of the *GH3* gene family which encodes an indole-3-acetic acid-amido synthase in rice, acts as an activator of disease resistance by transient and cell-specific suppression of auxin signaling [115]. Constitutive overexpression of *OsGH3.1* was shown to reduce auxin content and to enhance defense responses and resistance to *Magnaporthe grisae* in rice [111]. Thirty four defense genes were up- and down-regulated in the transgenic rice plants overexpressing *OsGH3.1* though no information were provided concerning phytoalexin gene induction in this case. Jones and colleagues have shown recently that the increased susceptibility to biotrophic pathogens and re-direction of phytoalexin metabolism observed in *Arabidopsis* plants overexpressing the *AFB1* gene (which encodes an auxin receptor and whose mRNA is partially resistant to a targeting auxin receptor- microRNA), were probably due to the negative effect of auxin on SA signaling [12]. Conversely, targeting of the auxin receptor mRNAs by overexpression of the microRNA miR393 resulted in direct suppression of auxin signaling and stabilization of inactive AUX/IAA-ARF, which negatively regulate auxin signaling through binding and inactivation of auxin response factors. Suppression of auxin signaling allowed the plant to mount a full SA response and to re-direct secondary metabolism towards the more fungitoxic phytoanticipins, glucosinolates [116] and away from the less fungitoxic phytoalexin camalexin.

The plant hormone abscisic acid (ABA) regulates the adaptive response of plants to environmental stresses such as drought, salinity, and chilling as well as biotic stresses *via* diverse physiological and developmental processes. As for auxins, it seems that ABA depressed fungal, bacterial and viral resistances of plants by negatively regulating genes involved in plant defense mechanisms though there are some reports of stress-induced elevated ABA concentrations being associated with decreased susceptibility to fungal pathogens, for instance in tomato challenged with *Phytophthora parasitica* [117]. Loss-of-function mutations in the ABA pathway led to ABA deficiencies and increase in disease resistance in tomato to, respectively, the necrotrophic fungus *B. cinerea* [118] and the pathogenic bacterium *Erwinia chrysanthemi* [119]. Exogenous applications of ABA made tobacco plants more susceptible to Tobacco Mosaic Virus (TMV) [120] and led to the down-regulation of genes encoding PR protein β-1,3-glucanase isoforms in tobacco cell cultures [121]. ABA was also involved in the regulation of phytoalexins. The synthesis of the bean (*Phaseolus vulgaris*) phytoalexin kievitone was downregulated by ABA [122]. ABA reduced glyceollin production in soybean as a response to *Phytophthora megasperma* [123] or *P. sojae* [124], as well. Rishitin and lubimin production, two phytoalexins from Solanaceae, was also found to be diminished by ABA in potato tubers infected by *P. infestans* [125].

To understand the mechanisms of the regulation of phytoalexin expression under the control of ABA, capsidiol biosynthesis was assessed in two tobacco (*N. plumbaginifolia*) nonallelic mutants, *Npaba2* and *Npaba1* deficient in ABA *vs.* wild-type plants as a response to abiotic (induction with cellulose or arachidonic acid) or biotic stresses [126]. The mutant *Npaba2* was impaired in zeaxanthin epoxidase (ZEP) and *Npaba1* was deficient in abscisic aldehyde oxidase. Total capsidiol phytoalexin accumulation in the two mutants doubled the amount of capsidiol in wild-type plants, even in those inoculated with *B. cinerea* which is known for its ability to produce ABA. This was the consequence of an enhanced expression of two genes acting on the capsidiol biosynthesis pathway, *5-epi-aristolochene synthase* (*EAS*) and *5-epi-aristolochene hydroxylase* (*EAH*). At the same time, the transcription activity of ZEP and 9-*cis*-epoxy-carotenoid dioxygenase which encode enzymes of the upstream pathway of ABA synthesis, decreased. ABA thus negatively regulates elicitor-induced phytoalexin production in tobacco. Induction of plant hormone catabolic pathways was also observed during capsidiol accumulation, being this catabolism not limited to ABA, but also affecting the gibberellins. In fact, both the expression of the gene encoding *ABA-8*′*-hydroxylase* (*ABAH*) and of that encoding *gibberellin-2-oxidase* (*GA-2Ox*) [127] were induced in parallel to the expression of *EAS* and *EAH* as well as the 2-fold increase in capsidiol. The expression pattern observed for *ABAH* and *GA-2Ox* implied a regulatory role of abscisic acid and likely gibberellins catabolic pathways on capsidiol accumulation in tobacco [126]. This clearly illustrates plant hormone cross talk for phytoalexin regulation.

There is little evidence of phytoalexin expression being regulated by gibberellins with the exception of the possible implication of gibberellin catabolism in the accumulation of capsidiol in tobacco (see above) [126]. Altered disease development was reported in rice plants overexpressing Elongated uppermost internode (*Eui*) gene encoding a gibberellin deactivating enzyme [128]. Conversely, knockout of this gene was found to compromise resistance to disease, indicating that gibberellins negatively regulate rice basal disease resistance. Although the induction of a PR1a pathogenesis-related protein was reported during pathogen resistance, no mention of a possible modulation of phytoalexins by gibberellins was made.

Cytokinins (CKs) are phytohormones involved in various regulatory processes during plant development [129]. Reports of the role of CKs in plant disease resistance are still scarce. Elevated CK levels were associated to resistance against viruses [130,131] and suppression of the HR in tobacco [132]. Plant-derived CKs were also shown to promote resistance of *Arabidopsis* to *Pseudomonas syringae* pv. *tomato DC3000* (*Pst*) via the salicylic acid response factor TAG3/Non-expressor of pathogenesis-related genes-1 (*NPR1*) [133]. Modulating CK levels or signaling activity in cytokinine-oxydase- or isopentenyl transferase-overexpressing plants in *ahk2 ahk3* mutants (double knockout of the CK receptor) correlated with altered resistance. Induction of phytoalexin synthesis upon resistance of *Arabidopsis* to *P. syringae* was not reported in this study.

More recently, CK overexpression was shown to enhance resistance against *P. syringae* pv *tabaci* in tobacco (*Nicotiana tabacum*) [100]. The CK-mediated resistance in tobacco strongly correlated with up-regulated synthesis of two major phytoalexins from that plant, the diterpenoid capsidiol and the coumarin scopoletin. Since CKs are aminopurine-derived compounds containing an isopentenyl moiety, the role of this phytohormone in the pathosystem between tobacco and *P. syringae* pv *tabaci* was addressed through the overexpression of the *isopentenyl transferase* (*ipt*) gene from *Agrobacterium tumefaciens* driven by the synthetic pathogen-inducible *4xJERE* promoter in the *4xJERE:ipt* plasmid. Isopentenyl transferase indeed catalyzes the rate-limiting reaction in the route to CKs.

In *ipt*-overexpressing tissues challenged with *P. syringae*, CK production was enhanced and this correlated well with increased resistance to this pathogen. As the protocol used for pathogen inoculation included pre-infiltration of leaves with *A. tumefaciens* which can interfere with symptom development, experiments were completed by two additional genetic approaches leading to ectopic production of CKs independent of prior infiltration with *A. tumefaciens*. The *ipt* gene was successively expressed under the control of a senescence-induced promoter (SAG12:ipt) and under the control of a chemically inducible tetracycline-dependent promoter (TET:ipt). In both transformed tobacco lines, there was a strong CK accumulation well correlating with disease symptom reduction.

Changes in endogenous CK concentrations enhanced the production of capsidiol and scopoletin by respectively 4.6 and 6.4-fold compared with the controls. Ectopic phytoalexin synthesis resulting from overexpression of the *4xJERE:ipt* construct was associated with the CK-mediated resistance against *P. syringae*. Interestingly, there was an induced expression of 5-*epi*-aristolochene synthase, catalyzing a rate-limiting step in the route to capsidiol, together with an induction of the cinnamate-4-hydroxylase which is implied in the phenylpropanoid pathway leading, namely, to the coumarin scopoletin. Conversely, expression of other enzymes related to phytoalexin biosynthesis such as the glucosyltransferase TOGT involved in the sorage of scopoletin (see section 3), phenylalanine ammonia lyase, the key enzyme of the phenylpropanoid pathway and flavonol synthase 1, were respectively moderate, not significantly affected or even repressed upon CK-mediated resistance of tobacco to *P. syringae*. Importantly, this CK-mediated resistance was also shown to be SA- and JA- independent.

## 5. Phytoalexin Accumulation in the Upregulation of Defense-Related Marker and Elicitor Genes

In the new concept of innate immunity for plants, defense responses are triggered after recognition by the plant cell of conserved Pathogen/Microbe-Associated Molecular Patterns (PAMPs/MAMPs) or by endogenous molecules released by pathogen ingress and called Danger-Associated Molecular Patterns (DAMPs) [134–136]. Early responses after elicitor perception are characterized by signaling processes including ion fluxes, Mitogen-Activated Protein Kinase (MAPK) cascade [137] and production of reactive oxygen species [138].

**Figure 5:**
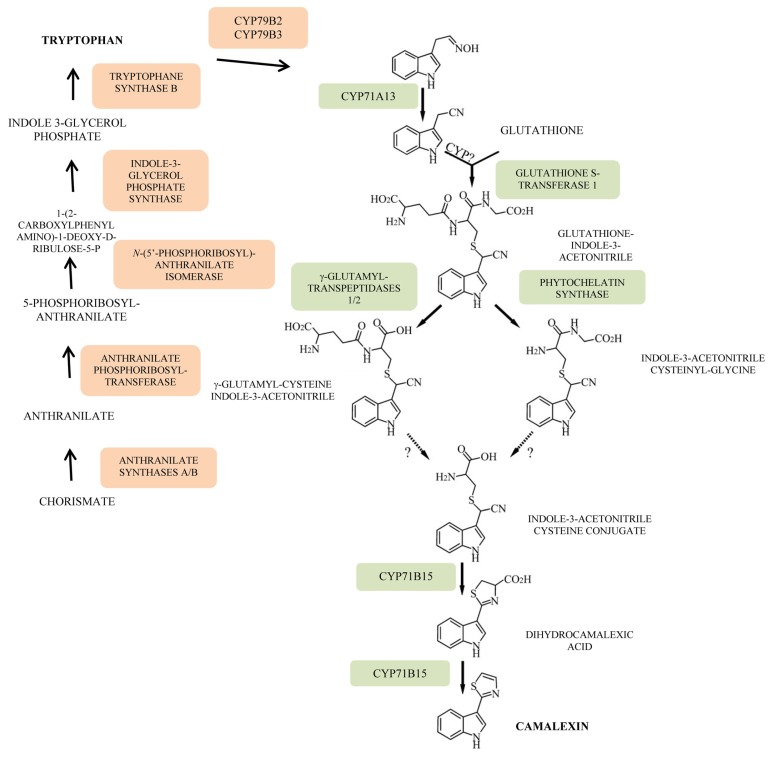
Biosynthetic pathways from chorismate to tryptophan and from tryptophan to camalexin (adapted from [139,140]).

In this context, MAPKs of *Arabidopsis*, respectively, MPK3 and MPK6, were shown to be activated upon MAMP treatment or following infection by a pathogen with correlated induction of the responses further downstream such as phytoalexin accumulation by using both gain-of-function and loss-of-function transgenic systems. [13]. MPK3 and MPK6 take part in a pathogen-responsive MAPK cascade, MAPKKKa/MEKK1-MKK4/MKK5-MPK3/MPK6, involved in the up-regulation of numerous enzymes of the biosynthetic route from chorismate to camalexin via tryptophan (Trp) [139,140] (Figure 5). Constitutively active mutants of the upstream MAPKKs, that is, tobacco NtMEK2DD and *Arabidopsis* MKK4DD or MKK5DD, were overexpressed under the control of a steroid-inducible promoter. Upon treatment with the steroid dexamethasone (DEX), induction of the active MAPKs, MPK3/MPK6 activated the endogenous MAPKs, which in turn induced the responses further downstream such as accumulation of the phytoalexin camalexin. Camalexin production was associated with the induction of numerous genes encoding enzymes acting on the biosynthesis of this phytoalexin, including the route from chorismate to Trp, on one hand, and the pathway from Trp to camalexin (Figure 5). Specifically, genes encoding anthranilate synthase a and b (ASA and ASB), phosphoribosylanthranilate transferase (PAT), indole-3-glycerolphosphate synthase (IGPS) and Trp synthase b (TSB), were all highly induced with levels of induction ranging from 12-fold (PAT) to more than 35-fold (TSB) 6h after DEX treatment. Genes encoding the P450 enzymes operating in the biosynthetic pathway from Trp to camalexin, namely the one catalyzing conversion of Trp to indole-3-acetaldoxime (*CYP79B2* gene) showed a 50-fold increase. Expression of P450 enzyme genes acting at the end of the pathway, for instance the *CYP71B15* (*PHYTOALEXIN DEFICIENT 3*, *PAD3*) gene encoding the multifunctional enzyme converting cysteine-3-indolacetonitrile to camalexin, increased by 400-fold (Figure 5).

Mutations in MPK3 and MPK36 impaired *B. cinerea*-induced camalexin production and disease resistance, providing loss-of-function evidence supporting the role of this MAPK cascade in phytoalexin biosynthesis upon pathogen infection. Camalexin accumulation was reduced by 50% in *mpk3* mutant, delayed in *mpk6* mutant and completely abolished in *mpk3*/*mpk6* double mutant, resulting in increased susceptibility to *B. cinerea*. All together, these experiments clearly showed that MPK3 and MPK6, two MAP kinases of *Arabidopsis* play key roles in camalexin biosynthesis and resistance to fungal pathogens. Induction of camalexin after MAPK activation was considered to be independent of ethylene [13].

Phytoalexin accumulation was reported in the upregulation of defense-related marker genes in rice plants (*Oryza sativa*) exhibiting spontaneous hypersensitive response upon overexpression of the *Oryza sativa* Accelerated Cell Death and Resistance 1 (*OsACDR1*) gene which encodes a putative Raf-like Mitogen-Activated Protein Kinase Kinase Kinase (MAPKKK) [141]. Two compounds, sakuranetin and momilactone A, respectively, flavanone and diterpenoid phytoalexins from rice had their concentrations increased by 4-fold and 32-fold following infection by the rice blast fungal pathogen *Magnaporthe grisea*, in *OsACDR1* overexpressing lines while their accumulation in vector control plants were nil or negligible. The fact that *OsACDR1* overexpressing plants exhibited enhanced disease resistance to *M. grisea* through a reduction in the ability of fungal appressoria to penetrate into plant cells could be indirectly related with phytoalexin accumulation in transgenic lines. Conversely, *Osacdr1* knock-out and OsACDR1-RNAi rice plants showed increased susceptibilities to the pathogen though no information regarding a possible relationship between the loss-of-function of *OsACDR1*, decreased-disease resistance and phytoalexin content were provided in this study.

Systemic Acquired Resistance (SAR) is a long lasting defense response that is induced by localized infection providing subsequent protection against a broad spectrum of pathogens [142]. Resistance to several fungal and bacterial diseases has been obtained by overexpressing the Non-expressor of Pathogenesis-Related genes-1 (*NPR1*) in many plant species. NPRs1 play a critical role in SAR [143] and validation of their involvement in various defense networks arose from a number of overexpression studies. For instance, overexpression of *NPR1* was shown to confer resistance to bacteria, viruses and fungi in rice, [144], tomato [145], wheat [146] and carrot [147]. Recently, resistance against various fungal pathogens and a reniform nematode was obtained in transgenic cotton overexpressing *Arabidopsis NPR1* (*AtNPR1*) [148]. The *AtNPR1*-expressing lines which displayed namely high level of resistance to *Verticillium dahliae* had corresponding high levels of gossypol, a phytoalexin from cotton and derivated terpenoids, forming part of the overall defense response that protects the *AtNPR1*-transformants from disease.

The Rac family also named Rop family belongs to the Ras superfamily of GTPases. This family constitutes one of the most important regulators of signal transduction in plants, participating to their adaptation to various environmental situations [149]. Rac proteins are also involved in innate immunity namely in rice and barley [149–151]. Importantly, the *Oryza sativa* Rac family OsRac1 forms a complex termed defensome with downstream proteins which regulate reactive oxygen intermediate (ROI) production, hypersensitive-like responses, lignin biosynthesis and phytoalexin accumulation as well as the transcription of pathogenesis-related genes [150,152]. Transgenic rice plants expressing the constitutively active OsRac1 exhibited HR-like responses and increased resistance both to a virulent race of the rice blast fungus and a virulent race of bacterial blight together with an enhancement of the production of the rice phytoalexin momilactone A (19- to 180-fold higher than the levels of untransformed rice plants) [150]. Phytoalexin content in the transgenic lines expressing the dominant-negative OsRac1 was similar to control plants.

Overexpression of an homologue of mamalian selenium-binding proteins, the rice *O. sativa* selenium-binding protein homologue (*OsSBP*) led to increased resistance to both rice blast fungus and rice bacterial blight *via* activation of plant defense mechanisms including accumulation of the rice phytoalexin, momilactone A [153]. Although there are some reports of the occurence of selenium binding proteins in plants, their functions remain unclear [154,155]. A full length *OsSBP* cDNA fragment was inserted in sense- or antisense-orientation under the control of the CaMV 35S promoter. *OsSBP* overexpressing rice lines exhibited delayed disease symptoms upon rice blast fungus while downregulated expression of *OsSBP* resulted in symptoms slightly more severe than in controls. Correspondingly, overexpression of *OsSBP* led to a 20–25-fold increase in momilactone A, being phytoalexin levels in *OsSBP* downregulated lines similar as those in the wild-type parental rice. Besides, there was also an induction in the PR gene expression as well as enhancement of H_2_O_2_ accumulation. The fact that overexpressing *OsSBP* rice plants also exhibited disease resistance to bacterial blight was consistent with the overall relevance of this protein in modulating the defense mechanisms of that plant.

Calcium regulates various cellular processes in plants as an ubiquitous internal second messenger, transferring signals received at the cell surface to the inside of the cell *via* spatial and temporal changes in its concentration. The information encoded within the Ca^2+^ transients is decoded and transmitted by a toolkit of Ca^2+^-binding proteins that regulate transcription *via* Ca^2+^-responsive promoter elements, regulating protein phosphorylation [156]. Several families of calcium sensors have been identified so far of which the best known are calmodulins (CaMs) and CaM-related proteins [157]. The second major class is the Ca^2+^-dependent protein kinases [158]. The third family consists of calcineurin B-like proteins (CBLs) acting as Ca^2+^ sensors to activate specific protein kinases, CBL interacting protein kinases (CIPKs) [159]. Since Ca^2+^ mobilization as a reponse to pathogen-associated molecular patterns plays a pivotal role in innate immunity in plants, the molecular links between this cation and downstream defense responses, namely phytoalexin production, were investigated using gain-of-function approaches and an RNAi strategy to address regulation of PAMP-induced defenses by two genes *OsCIPK14* and *OSCIPK15* encoding CIPKs in rice (*O. sativa*) cultured cells [160]. These two genes were rapidly induced in rice transgenic lines by microbe-associated molecular patterns, including chitooligosaccarides and a *Trichoderma viride*/ethylene-inducing xylanase (TvX/EIX). *OsCIPK14/15* overexpressing plants showed an increase in the two major phytoalexins of rice, momilactones and phytocassanes, as evidenced by LC-MS-MS analyses. This was the result of a slow and prolonged expression of genes encoding cyclase enzymes involved in the biosynthesis of these phytoalexins, namely, *ent*-copalyl diphosphate synthase 4 and *ent*-kaurene synthase-like 4 for momilactones and *ent*-copalyl diphosphate synthase 2 and *ent*-kaurene synthase -like 7 for phytocassanes, respectively. The TvX/EIX-induced phytoalexin biosynthesis was significantly suppressed in the *OsCIPK14/15*-*RNAi* lines.

Momilactones and phytocassanes are also regulated in rice by the *Oryza sativa* TGA factor for phytoalexin production 1 named *OsTGAP1* [161]. Transformed rice plants constitutively expressing *OsTGAP1* indeed showed enhanced accumulation of both phytoalexins. OstGAP1 regulates momilactone phytoalexin biosynthesis through positive control of the *OsKSL4* gene expression, responsible for the first committed step in the route to momilactone. Specifically, clustered genes for momilactone biosynthesis were shown to be coordinately regulated by OsTGAP1, leading to a hyper-accumulation of this phytoalexin as a response to elicitor treatment.

Camalexin biosynthesis in *Arabidopsis* was also shown to be regulated by microbial virulence factors of the Nep1-like protein family (NLP) [162]. Beside their role as toxin-like virulence factors, Nep1-like proteins (NLPs) act as signals triggering the plant innate immune responses involving a signaling cascade which includes reactive oxygen species and ethylene production, and ending in callose deposition, programme cell death and phytoalexin production [163,164]. Overexpression of a Nep1-like protein from *Pythium aphanidermatum* in *Arabidopsis* under the control of an ethanol-inducible promoter resulted in the strong transcriptional activation of genes working on the camalexin biosynthetic pathway (genes *ASA1* and *TSA*) (see above).

Activation-tagging is a powerful system for creating gain-of-function mutants in plants. Upon random introduction into the genome of a T-DNA containing enhancer sequences, there is an increased expression of neighboring genes on either side of it, resulting in a gain-of-function phenotype. A T-DNA tag was used to identify a neighbor gene overexpressed in a *Spotted leaf 18* mutant (*Spl18*) displaying strong resistance to blast disease and obtained by an activation tagging system in rice [165]. An ORF was located about 500 bp downstream of the inserted T-DNA and the deduced protein named OsAT1 presented a sequence similarity to an acyltransferase whose expression was induced in tobacco reacting hypersensitively. Combination of *OsAT1* genomic DNA downstream of a modified 35S promoter was introduced to rice. Overexpression of *OsAT1* induced several defense responses, including transcriptional activation of PR protein genes, accumulation of two phytoalexins from rice, sakuranetin and momilactone A, resulting in the plant resistance to blast disease and bacterial blight. Since the only one report of an acyltransferase being involved in the plant disease resistance mechanisms was *Arabidopsis* [166,167], it was unexpected that the overexpression of this acyltransferase could lead to upregulation of phytoalexins in rice.

We have seen in this section that, in addition to hormone signaling (section 4), phosphorylation relays and cascades, defense-related markers genes, calcium sensors and elicitors are also potentially important regulators for the modulation of phytoalexin production and pathogen resistance.

## 6. Phytoalexin Engineering: Where Plant and Human Interests do not Meet

As some phytoalexins, especially cottonseed and potato phytoalexins, display a certain level of toxicity for humans, there was a crucial interest in engineering those plants for abolishing accumulation of these undesirable compounds.

Genetic manipulation of plants for technological applications can affect phytoalexin expression. This is the case with potatoes which produce a number of antinutritional phytoalexins such as the steroid glycoalkaloids α-solanine and α-chaconine [168] (Figure 2). Potatoes genetically modified with an antisense potato invertase gene, exhibited a modified phytoalexin profile regarding glycoalkaloids. Specifically, transgenic lines were obtained by introducing a potato acid invertase gene sequence in antisense orientation driven by a patatin promoter and subtended by the *Agrobacterium*-derived nopaline synthase terminator. This was done to genetically manipulate through a reduction in the amount of invertase, the accumulation of reducing sugars responsible for subsequent browning problems during cooking at high temperatures. Such transformation namely decreased glycoalkaloid accumulation in potatoes *vs.* controls. It was speculated that diminutions in the carbohydrate pool in antisense invertase lines may reduce the UDP-glucose:solanidine glucosyltransferase and the UPD-galactose:solanidine galactosyltransferase-mediated conversion of solanidine to γ-chaconine and γ-solanidine, respectively, decreasing the amounts of the toxic glycoalkaloids α-chaconine and α-solanidine. Modifications of these toxic secondary metabolites in potatoes were related to a direct effect of the transgene rather than to interactions of the plant with its environment.

**Figure 6:**
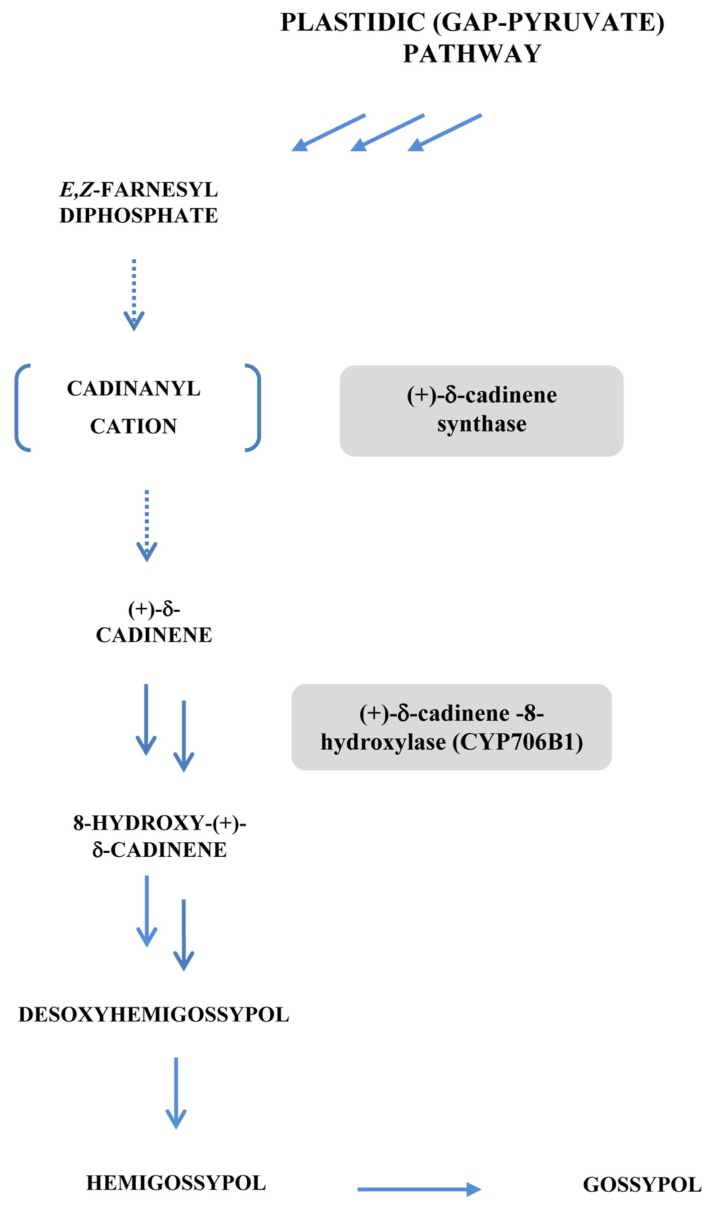
Scheme of biosynthetic pathway from farnesyl diphosphate to gossypol.

Gossypol and its congeners are dimeric sesquiterpene phytoalexins of the cadinane family from cotton (*Gossypium hirsutum*) deriving from the plastidic methylerythritol phosphate (GAP-pyruvate) pathway [161]. The latter leads to two C_5_ units, respectively isopentenyl-pyrophosphate (IPP) and its isomer dimethylallyl-pyrophosphate (DMAPP). Condensation of these two C_5_ units and larger IPP- and DMAPP-derived building blocks such as the C_10_ unit, geranyl pyrophosphate (GPP), the C_15_ unit, farnesyl pyrophosphate (FPP) and the C_20_ unit, geranylgeranylpyrophosphate (GGPP), yields monoterpenes, sesquiterpenes (such as gossypol), triterpenes (steroids), tetraterpenes (carotenoids) and diterpenes. The starter of the biosynthetic route to gossypol is farnesyl diphosphate (FPP) (Figure 6). In cotton, (+)-δ-cadinene synthase (CDNS) catalyzes the cyclization of *E*,*Z*-FPP to (+)-δ-cadinene, involving isomerization of FPP to a nerolidyl intermediate, the cyclization of which leads to a *cis*-germacradienyl cation. This is followed by 1,3-hydride shift, cyclization to a cadinanyl cation and deprotonation to form (+)-δ-cadinene [169,170]. CDNS catalyzes the first committed step in the route of the cadinane sesquiterpenoids from FPP, making this enzyme a crucial control component for gossypol engineering (Figure 6). According to the works of Chen and colleagues [171,172], four cDNAs of *cdn* synthase have been isolated from *Gossypium arboreum*, *i.e.*, *cdn*1-C1, *cdn*1-C14, *cdn*1-A and *cdn*1-C2.

Gossypol plays a role as a phytoalexin produced in response to the fungal pathogen *Verticillium dahlia* Kleb. and to bacterial blight, caused by *Xanthomonas campestris* pv. *malvacearum* (Smith) Dye. Beside its presence in the epidermis and hairs of developing roots together with its storage within the very typical lysigenous gossypol glands in the foliage, gossypol is the main cadinane sesquiterpenoid in developing and mature seeds as well as in the cottonseed glands. Gossypol is known to be toxic and to have antifertility effects on animals and humans ([173] and references therein) and must be removed from cottonseed oil prior to human consumption. To address this problem and for generating the trait of gossypol-free seeds in cultivated cotton species, genetic manipulation of the pathway leading to this compound through overexpression of antisense (+)-δ-cadinene synthase was carried out.

Two genetic approaches using *a priori* silencing strategy with the introduction of a (+)-δ-cadinene synthase gene, respectively, *cdn*-1-C1 from *G. arboretum* [171], and *cdn*-1-C4 from *G. Hirsutum* [174], in the antisense orientation were employed. The *cdn*-1-C1 from *G. arboretum* was found to be highly homologous to *cdn*-1-C4 from *G. Hirsutum* [174]. In the first set of experiments [171], cotton plants were harbored with a *cdn*-1C1 cDNA from *G. arboretum* in antisense transformation construct driven by the CaMV 35S promoter. Here, the expression of the antisense construct was sufficient to negate the transcripts from the expression of multiple constitutive *cdn* synthase genes, resulting in reduced CDN synthase activity and decreasing the cottonseed gossypol content by up to 70%. A new cotton variant with a markedly lowered gossypol content, but with no reduction in the number of lysigenous seed glands, was isolated by the same group from the progeny of hemizygous cotton plants (*G. hirsutum* cv. Coker 312) transformed with antisense *cdn*1-C1 cDNA [175]. Townsend and colleagues [174] made diametrically opposite observations in that the *cdn* synthase gene they used never affected gossypol levels in the seeds of transformants with either a constitutive 35S promoter or a seed-specific soybean lectin promoter driven-antisense construct, or in stems of the constitutive antisense lines challenged with *V. dahliae*. The converse situation was observed when cotyledons were subjected to infection with bacterial blight, *X. campestris*. From these observations it ensued that gossypol production in cotton implicates a complex system of differential regulation of the CDNS. Given the high DNA sequence conservation among the different *cdn* synthase gene families, the ability of the antisense *cdn*1-C4 constructs to silence only blight-induced CDNS genes and neither the cotyledons nor the stems infected by *V. dahlia*e was somewhat intriguing.

Due to equivocal and apparently contradictory data recovered from the use of the antisense δ-cadinene synthase gene strategy aiming at reducing gossypol amounts in cotton, the feasibility of a targeted RNAi-based approach for suppression of the δ-cadinene synthase gene in that plant with resultant, but tissue-specific disruption of gossypol accumulation, was clearly demonstrated [176]. A 604-bp sequence from a δ-cadinene synthase (*Cad*1-A gene) cDNA clone from a *G. hirsutum* staged-embryo library was chosen as the trigger sequence. Interestingly, the selected portion of the clone had 80.9% to 99.8% homology to other gene sequences of other δ-cadinene synthase genes from the diploid, *G. arboreum*, and the tetraploid, *G. hirsutum*, cotton plants, aiming at targeting all members of this gene family. This sequence was used to make an intron-containing hairpin (ihp) construct and transcription of the ihpRNA sequence was under the control of a highly seed-specific α-globulin B gene promoter from cotton [177]. *Agrobacterium*-mediated transformation of *G. hirsutum* cv. Coker 312 with the final hairpin vector allowed the gossypol concentration in seeds to be decreased by 99%. Fortunately, RNAi-mediated silencing remained confined to the tissues expressing the hairpin RNA-encoding transgene in cotton, and it was observed that the presence of the transgene did not reduce the phytoalexin content in foliage, floral organs and roots. Here, the RNAi approach coupled to a highly tissue-specific promoter made feasible a selective and significant diminution in the amounts of a given phytoalexin which is deleterious for human health, without affecting its levels and the defense responses in other organs of the plant.

A patent describing a method for reducing levels of gossypol in cottonseed was derived from these results [178]. This included selective inducing RNA gene silencing in the seeds of a transgenic cotton plant, to interfere with expression of the δ-cadinene synthase gene or the δ-cadinene-8-hydroxylase gene (encoding formation of 8-hydroxycadinene on the route to gossypol) (Figure 6) in cottonseed without substantially affecting expression of that gene in the foliage, floral parts, and roots of the plant.

## 7. Conclusions

Starting in the 1950’s, research on phytoalexins has begun with biochemistry and bio-organic chemistry, resulting in the determination of their structure, their biological activity as well as mechanisms of their synthesis and their catabolism by microorganisms. Elucidation of the biosynthesis of numerous phytoalexins has permitted the use of molecular biology tools for the exploration of the genes encoding enzymes of their synthesis pathways and their regulators. Genetic manipulation of phytoalexins requires *a priori* a sound knowledge of the genes involved in their biosynthesis and how accumulation of a given phytoalexin can be modulated. Success of the transformation will also depend on the inability of the pathogen to counteract the phytoalexin action. It is well known that phytopathogenic fungi, particularly, are able to metabolize the phytoalexins to which they are exposed. Engineering of fungal genes responsible for detoxification of phytoalexins in plants has pointed out their role in the interactions between plants and pathogens. For example, overexpression in hairy roots of pea (*Pisum sativum* L.) of a pisatin demethylating activity (PDA) from the pea-pathogen fungus *Nectria haematococca* reduced the amounts of pisatin. As a result, transgenic plant tissues with reduced capability to accumulate pisatin were found to be less resistant to fungal infection [85]. Also, it appears clearly evident that, in phytopathogenetic fungi, ATP-binding cassette (ABC) transporters, which may extrude plant defense products as well as fungicides, act as virulence factors, providing protection against defense compounds produced by the host. Many factors thus interplay which could affect the outcome of the interaction between plants and pathogens.

Unexpectedly, engineering phytoalexins for disease resistance in plants seems to have been limited to exploiting only a few phytoalexin biosynthetic genes, especially those encoding stilbenes and some isoflavonoids. The first example of a disease resistance resulting from foreign phytoalexin expression in a novel plant was published only in 1993 [65]. One can imagine that such a success would have opened the way for a ferment of activity in this area, but, if transformations were then operated to investigate the potential of stilbene biosynthetic genes to confer resistance to pathogens [59,60,62], strategy in engineering phytoalexins from other plant species did not receive as much applications as might be expected. As previously stated in this review, interest in secondary metabolite engineering deals with their implications in human health and disease. The extraordinary success obtained with resveratrol, the phytoalexin of Vitaceae, is linked to the fact that in this case, the engineered system requires a relatively simple genetic construct. Resveratrol is indeed obtained in one single step from *p*-coumaroyl-CoA and three malonyl-CoA units catalyzed by stilbene synthase. As these substrate precursor molecules are present throughout the plant kingdom, the introduction of a single gene is therefore sufficient to synthesize resveratrol in heterologous plant species. Except the case of resveratrol biosynthesis which appears to be very simple, other phytoalexins (isoflavonoids, terpenoids) are formed through very complex biosynthetic pathways. Engineering the entire pathway is not feasible and the problem is to choose the right enzyme catalyzing the limitant reaction of the given pathway. Moreover, modalities of the expression of genes encoding a given enzyme can be unpredictable. In fact, overexpression of 7-*O*-methyltransferase in alfalfa led to different regiospecificities, producing mainly 4′-*O*-methyl derivatives instead of the expected 7-*O*-methyl derivatives [83].

New techniques for metabolic engineering have thus to be exploited in the next years to come. Namely, methodologies for generating high-quality libraries of enzyme variants and novel high-throughput screening (HTS) technologies will open the way for the engineering of enzymes for the biosynthesis of various phytoalexins with potent biological activities. Specifically, HTS technologies can rapidly lead to the identification of genes which modulate a particular biosynthesis pathway. Gathering all genes encoding for a biomolecular pathway will allow the assembly of genetic constructs for the synthesis of a given phytoalexin.

Limitations in the strategy of engineering phytoalexins also arose from the fact that the obtained resistance is sometimes too weak since it is well known that phytoalexins are less phytotoxic than chemical fungicides [16]. This thus justifies indirect approaches which allow modulation of the accumulation of phytoalexin employing transcriptional regulators or components of upstream regulatory pathways (see above sections 4 and 5). For example, overexpression in *Arabidopsis* of the active MAPKs, MPK3/MPK6 activated the endogenous MAPKs, which in turn induced genes encoding the P450 enzymes operating in the biosynthetic pathway from tryptophan to camalexin, showing their activities a 50-fold increase or in some cases a 400-fold increase [13]. As a consequence, the data presented in this review show undoubtedly that indirect modulation of phytoalexin levels through transgenic approaches paves the way for the creation of novel plants with improved pathogen resistance traits.
